# Height of the Pleura above the Clavicle

**Published:** 1857-11

**Authors:** 


*Height of the Pleura above the Clavicle.*

Before the New York Academy of Medicine, Dr. Isaacs, Demonstrator of Anatomy in the University of New York, stated that in fourteen subjects out of one hundred, the *pleura* rose two inches and upwards above the superior margin of the clavicle. He even suggests the possibility that death attributed to the entrance of air into a vein in operations low down in the neck may have been caused by the surgeon having opened the pleural cavity.

We have, since writing the above notice, received this excellent paper in pamphlet form from the author, for which he will accept our thanks.—*Nash. Jour. of Med. and Surgery.*

